# Global Climate Change Adaptation Priorities for Biodiversity and Food Security

**DOI:** 10.1371/journal.pone.0072590

**Published:** 2013-08-21

**Authors:** Lee Hannah, Makihiko Ikegami, David G. Hole, Changwan Seo, Stuart H. M. Butchart, A. Townsend Peterson, Patrick R. Roehrdanz

**Affiliations:** 1 The Betty and Gordon Moore Center for Science and Oceans, Conservation International, Arlington, Virginia, United States of America; 2 Bren School of Environmental Science and Management, University of California Santa Barbara, Santa Bárbara, California, United States of America; 3 Bio-Protection Research Center, Lincoln University, Lincoln, Canterbury, New Zealand; 4 School of Biological and Biomedical Sciences, Durham University, Durham, United Kingdom; 5 Environmental planning Institute, Seoul National University, Gwanak-gu, Korea; 6 BirdLife International, Cambridge, United Kingdom; 7 University Distinguished Professor Biodiversity Institute, the University of Kansas, Lawrence, Kansas, United States of America; Pacific Climate Impacts Consortium, Canada

## Abstract

International policy is placing increasing emphasis on adaptation to climate change, including the allocation of new funds to assist adaptation efforts. Climate change adaptation funding may be most effective where it meets integrated goals, but global geographic priorities based on multiple development and ecological criteria are not well characterized. Here we show that human and natural adaptation needs related to maintaining agricultural productivity and ecosystem integrity intersect in ten major areas globally, providing a coherent set of international priorities for adaptation funding. An additional seven regional areas are identified as worthy of additional study. The priority areas are locations where changes in crop suitability affecting impoverished farmers intersect with changes in ranges of restricted-range species. Agreement among multiple climate models and emissions scenarios suggests that these priorities are robust. Adaptation funding directed to these areas could simultaneously address multiple international policy goals, including poverty reduction, protecting agricultural production and safeguarding ecosystem services.

## Introduction

The need for adaptation to climate change is an emerging focus of international policy [1]. The impacts of climate change are already being felt from the tropics (e.g., small island states, coral reefs) to the arctic (e.g., first nations communities) [2]. New international funding is being mobilized to meet these new challenges, but the committed funds are still likely an order of magnitude less than the medium-term needs [3]. As a result, recently committed funding needs to be prioritized to areas with exceptional need and/or the potential for impact in multiple sectors simultaneously [4].

Ecosystem-based Adaptation is the use of ecosystem services to help people adapt to climate change. Intact ecosystems provide clean water, shoreline protection, and other services in the face of climate change, providing adaptation options that are often cheaper and more enduring than technical or engineering solutions [5]. Maintaining intact ecosystems benefits both human adaptation to climate change and biodiversity. Understanding linkages between sectors is an important first step toward realizing these benefits.

Priorities linking ecosystems and food production are particularly important because human and natural responses to climate change are interwoven [6–8]. Impoverished farmers are likely to use diverse livelihood strategies that include diverse natural ecosystems and agro-ecosystems [9], and that benefit from a range of ecosystem services [10,11]. Individual species’ range shifts can break up plant and animal communities, altering ecosystem services critical to agriculture, such as watershed protection [12]. Decline in agricultural productivity due to climate change can lead to crop expansion into nearby natural habitats as farmers attempt to compensate for lost yield by increasing area under production. This may result in biodiversity loss, as well as undermining the very ecosystem services that support agriculture [13]. Understanding of these inter-dependencies under climate change is emerging [14–16]. Subsistence farmers most sensitive to climate change are in the tropics [16,17], as are the species most sensitive to climate change [14,18,19]. Critical areas in which the axes of agricultural and ecosystem responses to climate change intersect are beginning to be documented for individual regions [10,20].

Adaptation action in these areas could simultaneously enhance food security and maintain ecosystem function. This has policy relevance on multiple levels, as food security and ecosystem integrity are two of the three major benchmarks for climate change response established in the United Nations Framework Convention on Climate Change (UNFCCC), and make substantial contribution towards sustainable development, thus helping meet all three UNFCCC criteria [21]. However, studies examining the interlinkage of agriculture and ecosystem change on a global scale are lacking.

Here we model changes in food production and biodiversity to find areas in which adaptation needs are high in both sectors. Our models focus on 1) rainfed agriculture which supports many of the world’s poorest farmers [22] and 2) restricted-range bird species (i.e., historical range <50,000 km^2^) which are indicative of highly complex and biodiverse ecosystems [23,24]. We find multiple areas in which declining crop productivity and shifts in restricted-range species coexist, indicating possible synergies between ecosystem health and human well-being. We suggest that these areas are a valuable initial set of priorities for research and action in climate change adaptation.

## Materials and Methods

### Methods Summary

We assessed declines in agricultural suitability and in the distribution of restricted-range bird species resulting from climate change for 15 major rainfed staple crops and 1,263 restricted-range bird species, using five general circulation models (GCM). We modeled change in climate suitability for the rainfed subsistence crops that are of greatest importance for poor farmers in each region. Data on crop climatic tolerances were extracted from the EcoCrop Dataset [7]. Species distribution models (SDM) were used to simulate range changes in response to climate change for 1,263 restricted-range bird species using a global database of occurrence records [25]. Restricted-range bird species were chosen as modeling subjects as they represent unique elements in regional ecosystems, have small range sizes that make them vulnerable to climate change [26] and are used to define areas of high ecological priority such as endemic bird areas and global biodiversity hotspots [23,25]. Ecosystem and agricultural adaptation priorities were defined as regions in the upper quartile (>25%) in terms of species losing range or crops losing suitability under climate change. We then aggregated results to identify areas where these crop and ecosystem adaptation priorities are in close proximity. We indicated level of agreement between GCMs using an ensemble histogram approach in which level of agreement is indicated by mapped color intensity. Agricultural priorities, ecosystem priorities and cells with combined priority are mapped with GCM agreement indicated by five color increments corresponding to level of agreement among the five GCMs used.

### Species distribution modeling approach

Species distribution models (SDMs) simulate distributions of species based on the association of their current range with climatic variables, and may be used to simulate future ranges under climate change. Because many SDM methods are available, we tested the effect of a range of SDMs on our results. We implemented seven SDMs that have previously been shown to be effective in modeling the distributions of a range of taxa [27]: Maxent [28]; classification tree analysis (CTA); generalized additive models (GAM) [29]; generalized boosted regression models (GBR) [30]; generalized linear models (GLM) [31]; multivariate additive regression splines (MARS) [32], and random forests (RF) [33]. All SDMs except Maxent were developed within the R module BIOMOD [34]. Absence data for CTA, GAM, GBR, GLM, MARS, and RF were generated from individual species’ range maps, taking random points within a 500km buffer of known occurrences. The occurrence data were divided, 70% for model construction and 30% for model evaluation. To produce binary maps from SDM output, we used maximized training sensitivity plus specificity values for Maxent while the area under the curve (AUC) of the receiver operating characteristic (ROC) plot was used to determine optimal thresholds for the other SDMs.

We chose six variables as climatic inputs for the SDMs, including maximum and minimum temperature, precipitation seasonality, precipitation of the warmest quarter, growing degree days above 5°C (GDD), and an aridity index. Precipitation seasonality measures the variability of precipitation throughout the year, helping distinguish between tropical, Mediterranean and temperate climates. The aridity index incorporates the effects of both precipitation and temperature on moisture availability. These climatic variables may be directly relevant to the bird species being modeled (e.g., minimum temperature), the habitats on which these birds depend (e.g., GDD), or both.

We evaluated model performance by comparing SDM output with the range map of each species using specificity, sensitivity, Kappa [35], True Skill Statistic (TSS) [36], and Matthews’ correlation coefficient (MCC) [37]. Maxent models showed the best performance in all criteria except sensitivity. We constructed final global priorities using each individual SDM and found that regions identified were identical across all seven SDM variants, although the specific species range simulations varied. The results reported here are from the Maxent SDM.

### Restricted-range species distribution data

We used comprehensive unique georeferenced point localities from BirdLife International’s World Bird Database for restricted-range terrestrial bird species [25] as input for all SDMs. For quality control we omitted occurrence points that fell outside each species’ known extent of occurrence (EOO) [25], buffered by 20 km. EOO was determined by expert opinion informed by the point data augmented by information in the published literature (field guides, family monographs, national distribution atlases, specific inventories and surveys), observer records (e.g. from www.worldbirds.org), and data from the Global Biodiversity Information Facility (www.gbif.org). The points are of variable precision depending on date of acquisition, but typically accurate to within 1-2 km. Species with <13 point localities were excluded from the analysis because SDM reliability declines at low numbers of occurrence points. The resulting final data set included 1,263 species that were modeled for this analysis.

### Agricultural suitability modeling approach

Global suitability for the 15 agricultural crops (Table 1) was modeled using crop environmental requirements defined by FAO’s EcoCrop database [7]. EcoCrop defines optimal growing conditions for a crop relative to multiple climatic parameters. We considered a region suitable for a crop where conditions were at least 50% of optimal, based on a linear interpolation between the conditions for optimal growth and the absolute limit to growth for that crop as defined in EcoCrop (see Table S1). We used the Spatial Production Allocation Model, which delineates crops currently grown in individual regions, to select crops for modeling [38]. Only crops currently grown in a region were modeled, since the need for adaptation is best measured as decline in crops actually produced. This approach builds on global modeling of agricultural suitability using EcoCrop in Lane and Jarvis (2007) [39].

**Table 1:** Species used for EcoCrop modeling.

common name	Scientific name
Banana	*Musa spp*.1
Barley	*Hordeum vulgare* L.
Common bean	*Phaseolus vulgaris* L.
Sugar cane	*Saccharum* * spp*.2
Cassava	*Manihot esculenta* Crantz.
Maize	*Zea mays* L3
Millet	*Panicum* *miliaceum* L.
Ground nut	*Arachis* * spp*.4
Potato	*Solanum tuberosum* L.
Rice	*Oryza sativa* L.5
Sorghum	*Sorghum* *bicolor* (L.)
Soybean	*Glycine max* (L.) Merrill
Sugar beet	*Beta vulgaris L*
Sweet potato	*Ipomoea batatas* (L.) Lam.
Wheat	*Triticum* * spp*.6

Some crop species contained different species and varieties, so the model adapted the widest range of environmental requirement among species/varieties. The list of species used in the model appears below the table. Temperature and precipitation requirements representing the environmental range of possible/optimal crop growth are detailed in Table S1.

* 1 *Musa acuminata* Colla, *Musa acuminata* x *M* *. balbis* , *Musa* *balbisiana* Colla, *Musa* *halabanensis* Meijer, *Musa* *salaccensis* Zoll.

* 2 *Saccharum barberi* Jesweit, *Saccharum* *edule* Hassk., *Saccharum officinarum* L., *Saccharum robustum* Brandes, *Saccharum sinense* Roxb., *Saccharum* *spontaneum* L.

* 3 *Zea mays* L. *s.* mays, *Zea mays* v. amylacea Sturt, *Zea mays* v. ceratina Kulash, *Zea mays* v. everta Sturt, *Zea mays* v. indentata Sturt, *Zea mays* v. indurata Sturt, *Zea mays* v. *tunicata* Sturt

* 4 *Arachis* *glabrata* Benth., *Arachis hypogaea* L., *Arachis* *pintoi* Krap. & Greg., *Saccharum sinense* Roxb.

* 5 *Oryza sativa* L. *s.* japonica, *Oryza sativa* L. *s.* indica, *Oryza sativa* L *s.* javanica including both land and paddy cultivations

* 6 *Triticum aestivum* L., *Triticum* *durum* Desf.,

The environmental requirements for individual crops are defined in EcoCrop by optimal ranges of maximum and minimum temperature, precipitation, and length of growing season (Table S1). We used the full range of environmental requirements spanning all varieties of each staple crop. We included a proxy for CO_2_ fertilization by adjusting water use efficiency through a linear interpolation of experiment-derived efficiency gains for both C3 and C4 crops [40]. Crop production was considered ‘lost’ when a crop’s suitability declined >50% within a grid cell. We evaluated crop suitability loss in a global grid, and determined the ratio of crops losing suitability to the number of crops currently grown for each grid cell.

### Climate data

Projections of future climate were obtained from transient simulations of five GCMs: MIROC3.2 (medium resolution), CSIRO Mk3.5, BCM2.0, CCSM3.0, INMCM3.0 selected to represent a range of global mean temperature change projections and regional precipitation change patterns. All models were implemented under two emissions scenarios (A2 and B1) [41] and two future time periods (20 year averages centered on 2050 and 2090). For each GCM and emissions scenario, temperature and precipitation anomalies between present and future time periods were interpolated to 2.5 arc-minute horizontal resolution and added to historical values from the WorldClim 1950-2000 database to produce simulated future climates [42]. Regional and global priority areas remained the same in the 2050 and 2090 scenarios, and global priorities remained the same across A2 and B1 scenarios, so only 2090 A2 results are reported here.

### Identifying adaptation priority regions

Based on current climate and modeled future climate scenarios, we calculated the number of bird species that lose climatic suitability in each 2.5 arc-minute grid cell for each future time step. The upper quartile (top 25%) of cells in terms of total species losing range were categorized as ecosystem adaptation priority areas. Similarly, agricultural adaptation priority areas were defined based on the upper quartile of cells in number of crops lost. To identify combined adaptation priority areas, we overlaid a one-degree grid on the 2.5 arc-minute agricultural and ecosystem adaptation priority cells. The resulting one-degree cells that contained both agricultural and ecosystem adaptation priority areas were used to identify global and regional adaptation priority areas.

Global adaptation priorities were defined by high GCM agreement (4-5 GCMs) on co-occurrence of ecosystem and agricultural adaptation priority in individual one-degree cells, together with adjacent areas of agricultural or species priority. These global priority areas have large crop and species change in close proximity and are therefore good candidates for multi-purpose adaptation funding. Regional priorities are areas with lower GCM agreement (1-3 GCMs) on combined crop and species priorities along with adjacent areas of agricultural and species priority. These regional priorities may be important locally or warrant additional research to more firmly establish coincidence of agricultural and ecosystem change. Where two global priorities or a global priority and regional priority co-occurred within a national boundary or ecoregion, they were combined (e.g., Atlantic Coast of Brazil).

## Results

**Figure 1:**
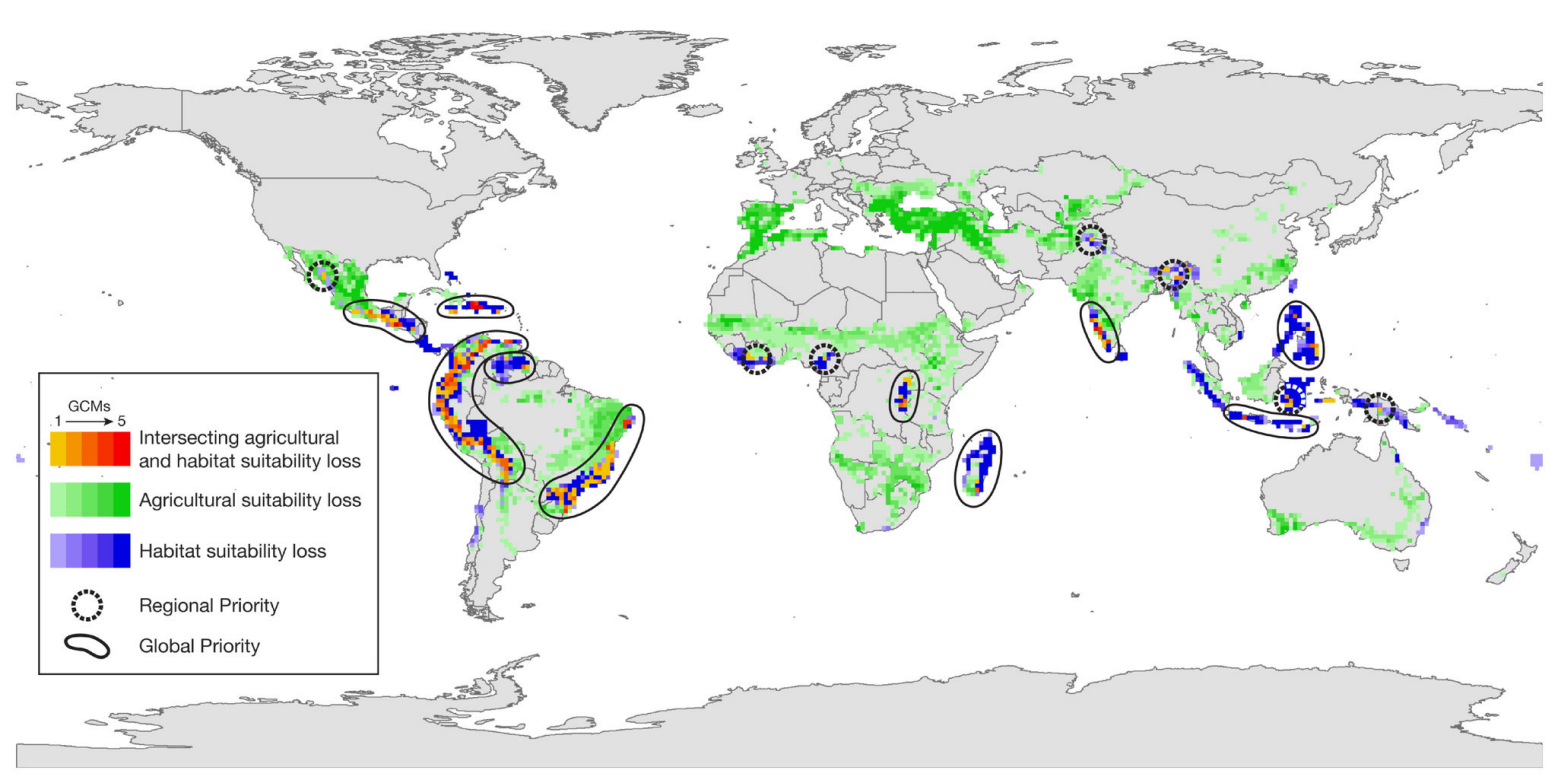
Global and regional priorities for adaptation of agriculture and biodiversity in the face of climate change. Global priorities (solid outlines) are areas of overlapping or contiguous agricultural and ecosystem change that appear by mid-century (2050). Regional priorities (broken circles) are areas of agricultural and ecological change that are less extensive and appear only later in the century (2090 scenarios). These global and regional priorities illustrated here are superimposed on a base map of 2090 A2 changes in both crops and restricted-range birds. Areas in which overall decreases are anticipated in crop suitability are shown in green, with increasing color intensity indicating multiple GCM agreement. Areas of declining climatic suitability for restricted-range birds are shown in blue, with increasing color intensity indicating multiple GCM agreement. Overlap of declining crop suitability and declining restricted-range bird climatic suitability is shown in shades of yellow (lowest GCM agreement) to red (highest GCM agreement).

Ten global priority areas for adaptation funding can be identified based on intersection of changes in agricultural suitability and climatic suitability for restricted-range species (Figure 1). The ten areas comprise 9.3% of the world’s habitable lands and 10.6% of remaining natural habitats; 7-9% of the world’s poor inhabit these areas and all of the areas intersect global biodiversity hotspots (Table 2). The agricultural and ecological changes that define these areas include declines in rainfed agricultural suitability encompassing 8.9% of the world’s arable land and 12.8% of currently cultivated land in the tropics and 69% of all modeled restricted-range bird species. Investment in these areas of intersecting suitability decline can help address the intertwined adaptation needs of agriculture and biodiversity.

**Table 2:** Human and ecological attributes of global and regional adaptation priorities.

Global Adaptation Priorities			crop loss (max %) *	bird suitability loss (%)**	area (km2)	population (1000)	poverty index (average)	global hunger index	Nations	# of global 200 ecoregions	global biodiversity hotspots
1		Central America	100.0	55.6	656,678	43,637	13.3	9.9	Mexico; Guatemala; Honduras; Nicaragua	3	Mesoamerica; Madrean pine-oak woodlands
2		Caribbean	100.0	54.0	96,575	22,603	28.0	18.6	Jamaica; Haiti; Dominica, Puerto Rico	2	Caribbean Islands;
3		Andes	100.0	44.9	2,931,172	93,053	12.0	7.4	Venezuela; Colombia/Ecuador; Peru; Bolivia; Argentina	17	Tumbes-Chocó-Magdalena Tropical Andes
4		Guiana Highlands	43.6	62.8	302,352	282	12.0	6.1	Venezuela	3	Tropical Andes
5		Atlantic Coast of Brazil	100.0	40.5	1,507,802	107,364	6.0	0.0	Brazil	3	Atlantic Forest
6		Albertine Rift	41.8	64.7	459,924	34,641	41.8	26.0	Zaire; Burundi; Tanzania; Uganda	6	Eastern Afromontane
7		Madagascar	93.4	78.0	587,041	16,194	35.0	27.5	Madagascar	4	Madagascar and the Indian Ocean Islands
8		Western Ghats	85.3	50.7	328,376	107,074	22.0	13.2	India	1	Western Ghats Sri Lanka
9		Philippines	80.8	62.2	299,764	75,257	15.0	13.0	Philippines	3	Philippines
10		Java	22.1	63.3	128,297	134,844	16.0	13.2	Indonesia	3	*Wallacea*
Regional Priorities												
r1		Northwest Mexico	80.0	66.7	149,589	2,234	4.0	<5	Mexico	3	Mesoamerica; Madrean pine-oak woodlands
r2		West Africa	15.4	77.8	337,507	18,655	14.0	14.0	Ivory Coast	1	Guinean Forests of West Africa
r3		Cross river	22.5	64.3	129,285	6,745	15.5	17.7	Cameroon; Nigeria	3	Guinean Forests of West Africa
r4		Western Himalayas	49.2	52.5	128,608	22,550	23.0	19.1	Pakistan	2	Himalaya
r5		Eastern Himalaya	68.7	60.0	582,384	68,840	21.7	22.4	India; Myanmar; Bhutan (Bangladesh)	6	Himalaya
r6		Sulawesi	19.6	75.8	174,600	16,366	16.0	13.2	Indonesia	2	*Wallacea*
r7		New Guinea	74.9	62.2	786,000	6,721	16.0	13.2	Papua New Guinea, Indonesia	6	Himalaya; Indo-Burma
	* Maximum % crop loss denotes the highest crop loss in any cell in the region. ** Cumulative % restricted-range bird loss of climatic suitability is the sum of total suitable area across all species in 2090 as a percentage of the sum of total suitable area across all species in 2000.											

The ten global adaptation priority regions and seven regional priorities are shown with their respective decline in crop suitability, decline in restricted-range bird suitability, area, population, development indices, and conservation indices. Maximum % crop loss denotes the highest aggregate loss in suitability across all crops in any individual 2.5 arc minute cell in the region (maximum loss is presented rather than average because many cells in each region show no change, resulting in uniformly low average loss values). Cumulative % restricted-range bird habitat loss is the sum of total suitable climate space across all species in 2090 as a percentage of the sum of total suitable habitat across all species in 2000. Development indices are the poverty index (PI) and the Global Hunger Index (GHI) of the International Food Policy Research Institute (IFPRI) [53]. PI is calculated from measures of health, education and standard of living; for reference, the average PI of OECD nations is 12.0. GHI is based on measures of undernourishment, the proportion of underweight children and early childhood mortality; GHI values of above 5 indicate ‘moderate hunger’, above 10 ‘serious hunger’ and above 20 ‘alarming hunger’ respectively. Population is derived from Global Rural-Urban Mapping Project version One (GRUMPv1) Adjusted Population Count Grid data for the year 2000 [54].

An additional seven regional priority areas harbor both agricultural and natural change that is less certain (i.e. less GCM agreement) than in the ten global priorities and therefore represent research priorities for more detailed regional analysis. These areas are Northwest Mexico, West Africa, the Cross River region, the Western Himalayas, the Eastern Himalayas, Sulawesi, and New Guinea. Most of these areas experience upper quartile crop loss or habitat suitability loss under most GCM scenarios, but with little inter-GCM agreement on areas of overlap.

The analyses used to derive these priorities are robust to alternative analytical methods, climate scenarios and climate models. The ensemble histogram approach used here (see Figures S1-S6) [43] presents the degree of agreement across multiple GCMs. The orange-red color ramp in Figure 1 indicates areas in which combined crop and ecosystem adaptation priorities are found in all five GCM results (red) through decreasing GCM agreement to combined crop and ecosystem priority found in only one GCM result (yellow). GCM agreement on non-overlapping agricultural and ecosystem adaptation priorities are shown in color ramps of green (crop loss) and blue (species range loss). This histogram approach highlights priorities that emerge repeatedly across multiple climate models, and are thus more robust to GCM uncertainty [43]. This ensemble histogram [43], in which GCM agreement is explicitly represented, contrasts with the ensemble mean approach in which the average of GCM results is taken. The ensemble histogram maintains relationships between climatic variables within individual GCMs, allowing representation of variable interactions that might be lost in an ensemble mean, for instance between temperature and precipitation in determining soil moisture. We have tested the ensemble histogram against ensemble mean approaches (Figures S4 and S5), and found that ensemble mean priorities are a subset of those determined using ensemble histograms. The priorities in Central and South America are robust across both methods, while the ensemble mean shows few priorities in Africa or Asia at the thresholds used here.

Uncertainty associated with species and agricultural modeling is more difficult to constrain. In the tropics, species ranges may not sample all climatic conditions that are physiologically tolerable, resulting in over-estimation of range loss. Adaptation priorities for non-avian restricted-range species may not match those for birds, due to different taxonomic climatic tolerances. SDM are fitted to empirical data using current climatic conditions and may not simulate future range changes well when future climatic conditions are outside the range of current climate. Adaptation in agriculture may moderate crop losses, resulting in over-estimation of crop impacts. For instance, consumption may switch to crops which may be grown in a region but are not culturally favored, once suitability for preferred crops declines. For these reasons, regional analyses incorporating social factors, taxa other than birds, and analyses of adaptation capacity are essential complements to this global analysis.

## Discussion

The priorities identified here reinforce and expand on the findings of other research into agricultural and biodiversity responses to climate change. Areas of agricultural vulnerability identified in this study overlap with those defined in other global assessments of agricultural impacts of climate change [44–46]. For instance, the Indian sub-continent emerges as an area of agricultural decline in both our results and in those of other studies [22,46]. Ecologically vulnerable areas identified in our restricted-range bird modeling are broadly consistent with impacts projected in several recent studies [47–49].

Possible adaptation responses in these priority areas might include actions that integrate adaptation for agriculture and ecosystems. For instance, agroforestry can help reduce soil temperatures and maintain soil moisture as air temperatures increase, and, with the appropriate choice of tree species, can help provide habitat for restricted-range birds and other wildlife. Protecting upland forests that provide fog interception or provide watershed protection for dams may be critical for agriculture in areas of declining precipitation, at the same time providing habitats that will permit range migrations in forest birds responding to climate change. Many of these solutions require planning on scales broader than individual farms or protected areas, which is why we have aggregated our results at spatial scales roughly coincident with scales of ecosystem and regional planning (one degree).

It is likely that finer-grained analyses will confirm some or all of the regional priority areas identified (dashed outlines, Figure 1) as regions of considerable change in both agriculture and ecosystems. For instance, in this study, the island of New Guinea (Indonesian Papua and Papua New Guinea) showed high change in restricted-range birds but little agricultural change. However, this region has very limited agricultural data because the subsistence crops prevalent there are poorly captured in global agricultural records. It is likely that improved agricultural data would reveal high adaptation needs in agriculture in this area, making it a joint priority for both human and natural adaptation.

The global adaptation priorities defined here have important policy relevance. First, they highlight areas in which it is most critical to address cross-sector inter-dependencies to achieve lasting adaptation results. Second, they illustrate a broader set of solutions to planning problems that must balance short-term human adaptation needs with long-term ecological sustainability. Finally, they help guide investment toward regions that may serve as tests for planning and adaptation solutions that can evolve to help other regions simultaneously meet multiple objectives of international agreements.

The UNFCCC holds as its goal the prevention of dangerous anthropogenic interference in the climate system, “within a time-frame sufficient to allow ecosystems to adapt naturally to climate change, to ensure that food production is not threatened and to enable economic development to proceed in a sustainable manner” [21,50]. Our results show that food production and ecosystem integrity are jointly vulnerable in ten world regions. Because global political progress on climate change mitigation is evolving slowly at present [51], it is probable that adaptation actions in these regions will be the only means of averting the kind of damage to human and natural systems that the world has committed to avoid in the climate convention.

Adaptation investments in these regions will, at the same time, realize progress towards achieving Millennium Development Goals and the goals of the Convention on Biological Diversity. The global and regional adaptation priority regions combined hold over 10% of the world population living in poverty, typically at less than half of the global average Human Development Index (Table 2). Targeting these areas can help meet the need for global adaptation funding priorities, as well as address poverty alleviation. The biodiversity in these areas is of global importance, as all are recognized in one or more global conservation priority ranking systems (e.g., hotspots, Global 200 ecoregions – see Table 2). Investment in these areas can help pioneer demonstrations that human development and ecological sustainability are intertwined - even more intimately than at present - as climate changes.

Designing adaptation investment strategies to meet these cross-sectoral goals requires decisions at multiple scales. This analysis provides a first set of global investment priorities. At the local scale, investments that capitalize on these opportunities may come through land sharing, in which case single investments can address both priorities, or through land sparing, in which case separate investments (each with strong links to the other sector) will be required. Both approaches can be effective [52], so wise investment requires sufficient understanding of local social and biological circumstances to know which will be most effective.

Adaptation practice is currently being pioneered in first-generation projects around the world. These efforts are addressing the adaptation needs of communities and ecosystems, increasingly recognizing deep interdependencies between the two. Meeting short-term development needs while establishing long-term sustainability of ecosystem services is a fundamental challenge. Among the best places to build experience in melding human and ecosystem responses will be regions experiencing substantial change in both. Innovative solutions and lessons from these areas can inform second-generation adaptation responses worldwide. Investment in the priority regions identified here is therefore a first step in developing a lasting, multi-sector approach to adaptation.

## Supporting Information

Table S1 **Species and parameters used for EcoCrop modeling.** Some crop species contained different species and varieties, so the model adapted the widest range of environmental requirement among species/varieties. The list of species used in the model appears below the table. Minimum and maximum temperature/precipitation requirements represent the environmental range where crop growth is possible and optimal minimum and maximum temperature/precipitation requirements are the environmental range of optimal growth.
(DOCX)

Figure S1 **Performance of each SDM tested by overall accuracy, Kappa, TSS, MCC, specificity and sensitivity.** Maxent scored highest performance except sensitivity that RF scored the highest performance.
(TIF)

Figure S2 **Global map of crop suitability and suitability for range restricted birds change in 2050 under A2 emission scenario.** Areas in which overall decreases are anticipated in crop suitability are shown in green, with increasing color intensity indicating multiple GCM agreement. Areas of declining climatic suitability for restricted range birds are shown in blue, with increasing color intensity indicating multiple GCM agreement. Overlap of declining crop suitability and declining restricted range bird climatic suitability is shown in shades of yellow (lowest GCM agreement) to red (highest GCM agreement).
(TIF)

Figure S3 **Global map of crop suitability and suitability for range restricted birds change in 2050/2090 under B1 emission scenario.** Areas in which overall decreases are anticipated in crop suitability are shown in green, with increasing color intensity indicating multiple GCM agreement. Areas of declining climatic suitability for restricted range birds are shown in blue, with increasing color intensity indicating multiple GCM agreement. Overlap of declining crop suitability and declining restricted range bird climatic suitability is shown in shades of yellow (lowest GCM agreement) to red (highest GCM agreement).
(TIF)

Figure S4 **Global map of crop suitability and suitability for range restricted birds change in 2050/2090 under A2 emission scenario using the ensemble mean of 5 GCMs.** Areas in which overall decreases are anticipated in crop suitability are shown in green. Areas of declining climatic suitability for restricted range birds are shown in blue. Overlap of declining crop suitability and declining restricted range bird climatic suitability is shown in red.
(TIF)

Figure S5 **Global map of crop suitability and suitability for range restricted birds change in 2050/2090 under B1 emission scenario using mean of 5 GCMs.** Areas in which overall decreases are anticipated in crop suitability are shown in green. Areas of declining climatic suitability for restricted range birds are shown in blue. Overlap of declining crop suitability and declining restricted range bird climatic suitability is shown in red.
(TIF)

Figure S6 **Global map of crop suitability and suitability for range restricted birds change in 2090 under A2 emission scenario using 6 Species Distribution Model results.** Areas in which overall decreases are anticipated in crop suitability are shown in green, with increasing color intensity indicating multiple GCM agreement. Areas of declining climatic suitability for restricted range birds are shown in blue, with increasing color intensity indicating multiple GCM agreement. Overlap of declining crop suitability and declining restricted range bird climatic suitability is shown in shades of yellow (lowest GCM agreement) to red (highest GCM agreement).
(TIF)
