# Supplementary material for: Global Climate Change Adaptation Priorities for Biodiversity and Food Security
Source: PLoS One. 2013 Aug 21;8(8):e72590. doi: 10.1371/journal.pone.0072590 (PMC3749124; doi:10.1371/journal.pone.0072590)
Supplement: Table S1 — Species and parameters used for EcoCrop modeling. Some crop species contained different species and varieties, so the model adapted the widest range of environmental requirement among species/varieties. The list of species used in the model appears below the table. Minimum and maximum temperature/precipitation requirements represent the environmental range where crop growth is possible and optimal minimum and maximum temperature/precipitation requirements are the environmental range of optimal growth. (DOCX) [file pone.0072590.s001.docx]

Table S1. Species and parameters used for EcoCrop modeling. Some crop species contained different species and varieties, so the model adapted the widest range of environmental requirement among species/varieties. The list of species used in the model appears below the table. Minimum and maximum temperature/precipitation requirements represent the environmental range where crop growth is possible and optimal minimum and maximum temperature/precipitation requirements are the environmental range of optimal growth.

| common name | scientific name | min. growing days | max. growing days | lethal  temperature | min temp. req. | opt. min. temp. req. | opt. max. temp. req. | max. temp. req. | min. prec. req. | opt. min. prec. req. | opt. max. prec. req. | max. prec. req. | improvement rate for water use |
| --- | --- | --- | --- | --- | --- | --- | --- | --- | --- | --- | --- | --- | --- |
| Banana | *Musa spp.* *1 | 180 | 365 | 0 | 12 | 23 | 35 | 42 | 650 | 1200 | 3600 | 5000 | 0.120 |
| Barley | *Hordeum vulgare* L. | 90 | 240 | -4 | 2 | 15 | 20 | 40 | 200 | 500 | 1000 | 2000 | 0.080 |
| Common bean | *Phaseolus vulgaris* L. | 50 | 270 | 0 | 7 | 16 | 25 | 32 | 300 | 500 | 2000 | 4300 | 0.120 |
| Sugar cane | *Saccharum spp.* *2 | 90 | 365 | -2 | 12 | 20 | 37 | 41 | 750 | 1000 | 3000 | 5000 | 0.080 |
| Cassava | *Manihot esculenta* Crantz. | 180 | 365 | 7 | 10 | 20 | 29 | 35 | 500 | 1000 | 1500 | 5000 | 0.120 |
| Maize | *Zea mays* L *3 | 65 | 365 | 0 | 10 | 16 | 33 | 47 | 400 | 600 | 1500 | 1800 | 0.080 |
| Millet | *Panicum miliaceum* L. | 55 | 280 | 0 | 15 | 20 | 32 | 45 | 200 | 500 | 750 | 1000 | 0.100 |
| Ground nut | *Arachis spp.*. *4 | 90 | 300 | 0 | 10 | 20 | 32 | 45 | 400 | 600 | 2000 | 4000 | 0.120 |
| Potato | *Solanum tuberosum* L. | 90 | 160 | -1 | 7 | 15 | 25 | 30 | 250 | 500 | 800 | 2000 | 0.125 |
| Rice | *Oryza sativa* L. *5 | 80 | 240 | 0 | 10 | 20 | 35 | 38 | 750 | 1000 | 2000 | 4000 | 0.080 |
| Sorghum | *Sorghum bicolor* (L.) | 90 | 300 | 0 | 8 | 22 | 35 | 40 | 300 | 500 | 1000 | 3000 | 0.140 |
| Soyabean | *Glycine max* (L.) Merrill | 75 | 180 | 0 | 10 | 20 | 33 | 38 | 450 | 600 | 1500 | 1800 | 0.120 |
| Sugar beet | *Beta vulgaris L* | 160 | 200 | -2 | 4 | 15 | 25 | 35 | 500 | 650 | 900 | 1200 | 0.120 |
| Sweet potato | *Ipomoea batatas* (L.) Lam. | 80 | 170 | 1 | 10 | 18 | 28 | 38 | 500 | 750 | 1250 | 5000 | 0.125 |
| Wheat | *Triticum spp.* *6 | 90 | 250 | 0 | 5 | 15 | 25 | 30 | 300 | 500 | 900 | 1600 | 0.050 |
|  |  |  |  |  |  |  |  |  |  |  |  |  |  |
| *1 *Musa acuminata* Colla, *Musa acuminata x M. balbis*., *Musa balbisiana* Colla, *Musa halabanensis* Meijer*, Musa salaccensis* Zoll. | | | | | | | |  |  |  |  |  |  |
| *2 *Saccharum barberi* Jesweit, *Saccharum edule* Hassk., *Saccharum officinarum* L., *Saccharum robustum* Brandes*, Saccharum sinense* Roxb., *Saccharum spontaneum* L. | | | | | | | | | | |  |  |  |
| *3 *Zea mays* L. s. mays, *Zea mays v. amylacea* Sturt*, Zea mays v. ceratina* Kulash, *Zea mays v. everta* Sturt*, Zea mays v. indentata* Sturt, *Zea mays v. indurata* Sturt, *Zea mays v. tunicata* Sturt | | | | | | | | | | | | | |
| *4 *Arachis glabrata* Benth*., Arachis hypogaea* L., *Arachis pintoi* Krap.& Greg., *Saccharum sinense* Roxb. | | | | | | |  |  |  |  |  |  |  |
| *5 *Oryza sativa* L. s. *japonica,* *Oryza sativa* L. s. *indica*, *Oryza sativa* L *s. javanica* including both land and paddy cultivations | | | | | | | | |  |  |  |  |  |
| *6 *Triticum aestivum* L., *Triticum durum* Desf., | | |  |  |  |  |  |  |  |  |  |  |  |
