# Supplementary figures and images for: Global Climate Change Adaptation Priorities for Biodiversity and Food Security
Source: PLoS One. 2013 Aug 21;8(8):e72590. doi: 10.1371/journal.pone.0072590 (PMC3749124; doi:10.1371/journal.pone.0072590)

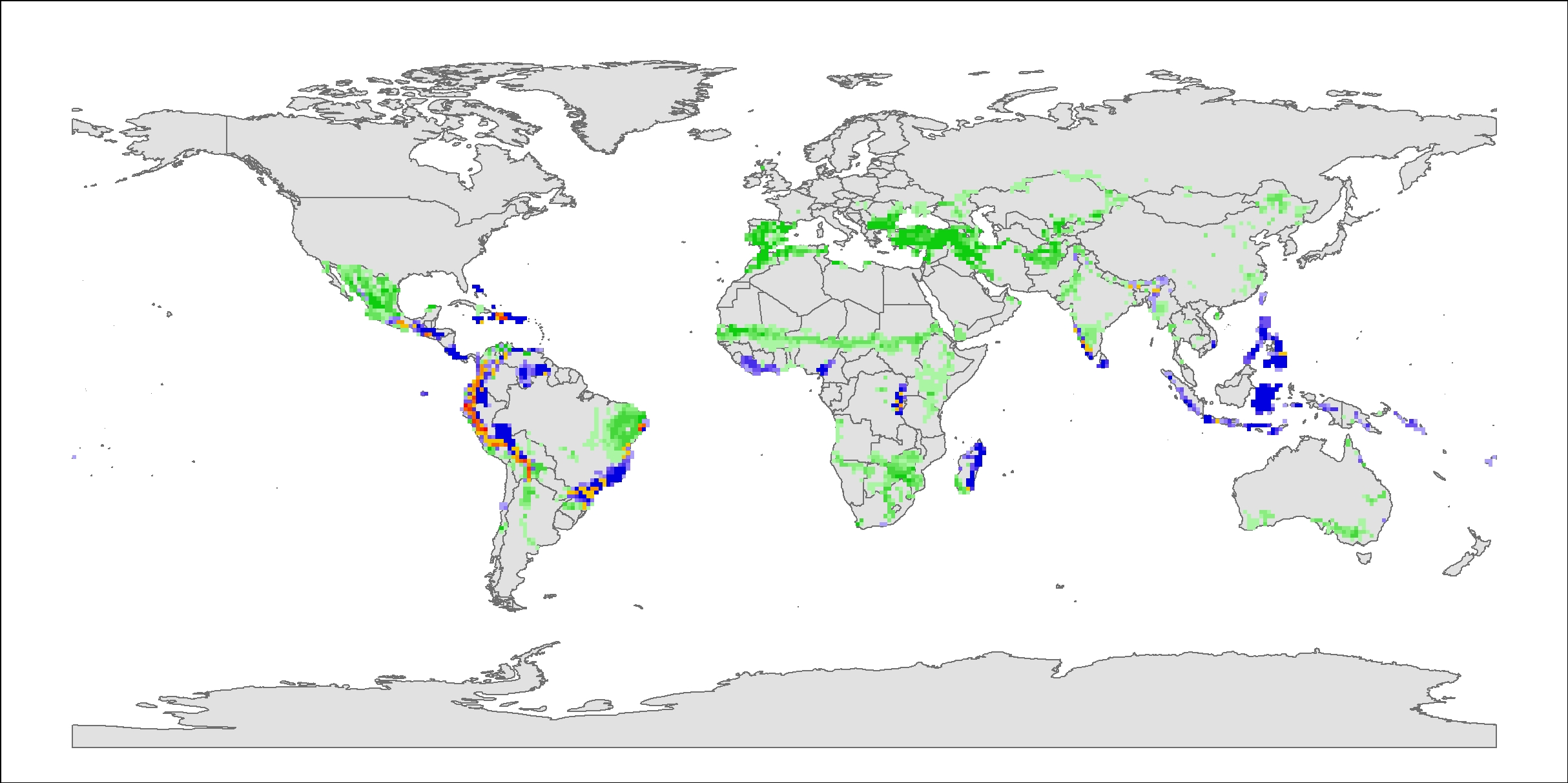

Supplement: Figure S2 — Global map of crop suitability and suitability for range restricted birds change in 2050 under A2 emission scenario. Areas in which overall decreases are anticipated in crop suitability are shown in green, with increasing color intensity indicating multiple GCM agreement. Areas of declining climatic suitability for restricted range birds are shown in blue, with increasing color intensity indicating multiple GCM agreement. Overlap of declining crop suitability and declining restricted range bird climatic suitability is shown in shades of yellow (lowest GCM agreement) to red (highest GCM agreement). (TIF) [file pone.0072590.s003.tif]

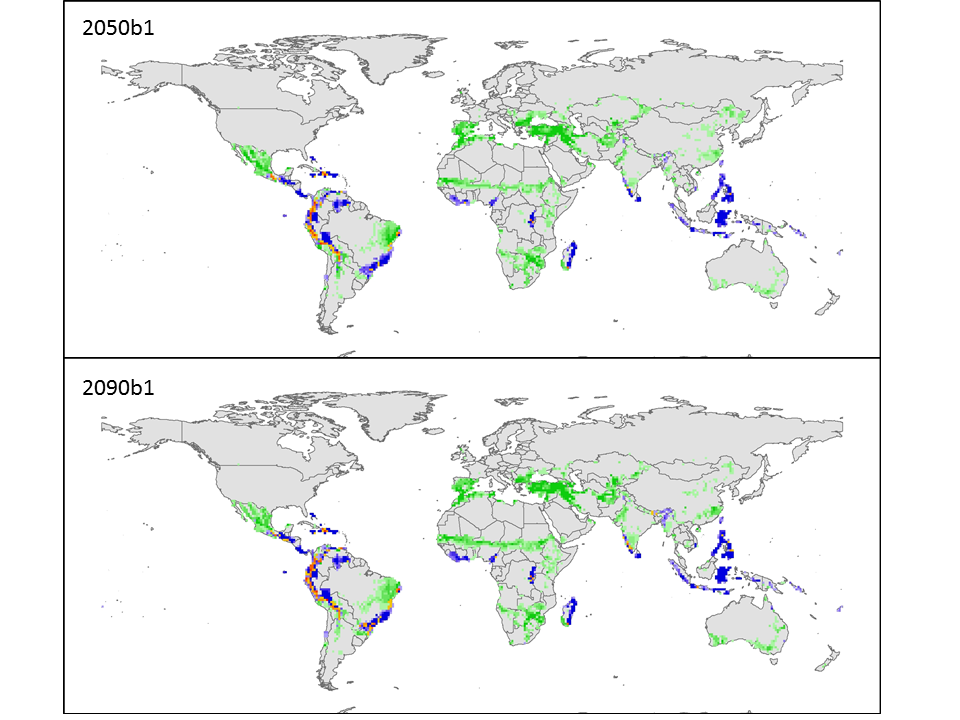

Supplement: Figure S3 — Global map of crop suitability and suitability for range restricted birds change in 2050/2090 under B1 emission scenario. Areas in which overall decreases are anticipated in crop suitability are shown in green, with increasing color intensity indicating multiple GCM agreement. Areas of declining climatic suitability for restricted range birds are shown in blue, with increasing color intensity indicating multiple GCM agreement. Overlap of declining crop suitability and declining restricted range bird climatic suitability is shown in shades of yellow (lowest GCM agreement) to red (highest GCM agreement). (TIF) [file pone.0072590.s004.tif]

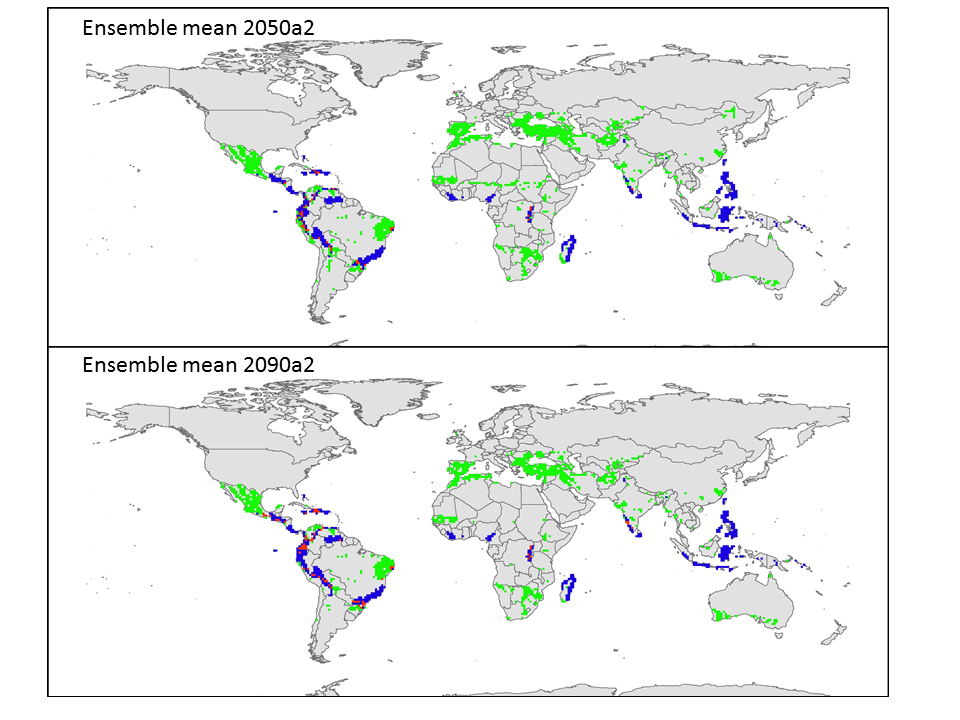

Supplement: Figure S4 — Global map of crop suitability and suitability for range restricted birds change in 2050/2090 under A2 emission scenario using the ensemble mean of 5 GCMs. Areas in which overall decreases are anticipated in crop suitability are shown in green. Areas of declining climatic suitability for restricted range birds are shown in blue. Overlap of declining crop suitability and declining restricted range bird climatic suitability is shown in red. (TIF) [file pone.0072590.s005.tif]

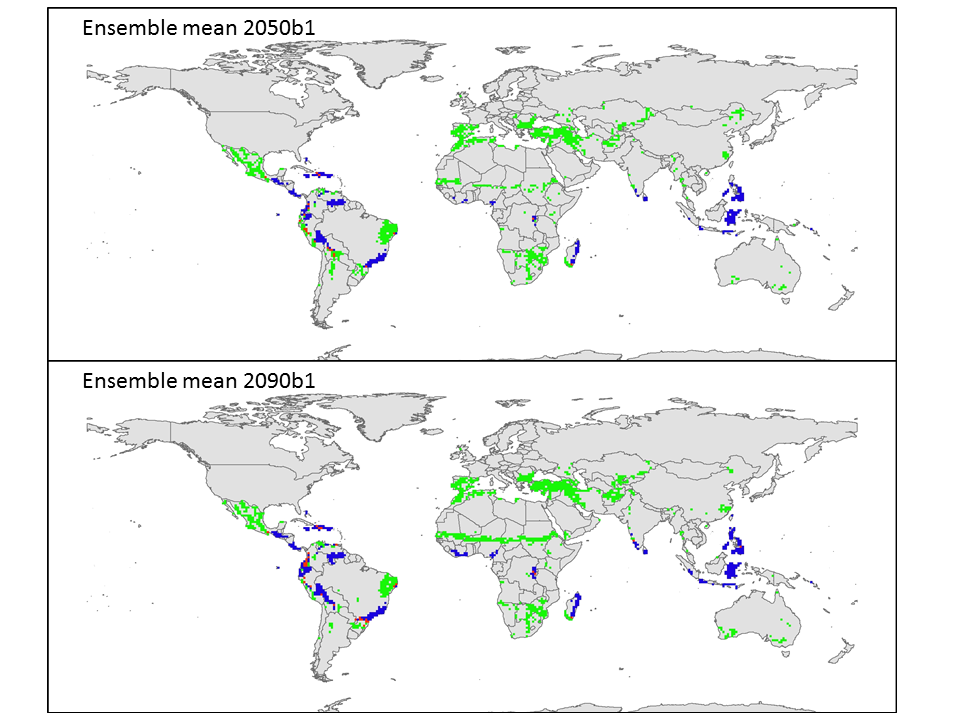

Supplement: Figure S5 — Global map of crop suitability and suitability for range restricted birds change in 2050/2090 under B1 emission scenario using mean of 5 GCMs. Areas in which overall decreases are anticipated in crop suitability are shown in green. Areas of declining climatic suitability for restricted range birds are shown in blue. Overlap of declining crop suitability and declining restricted range bird climatic suitability is shown in red. (TIF) [file pone.0072590.s006.tif]

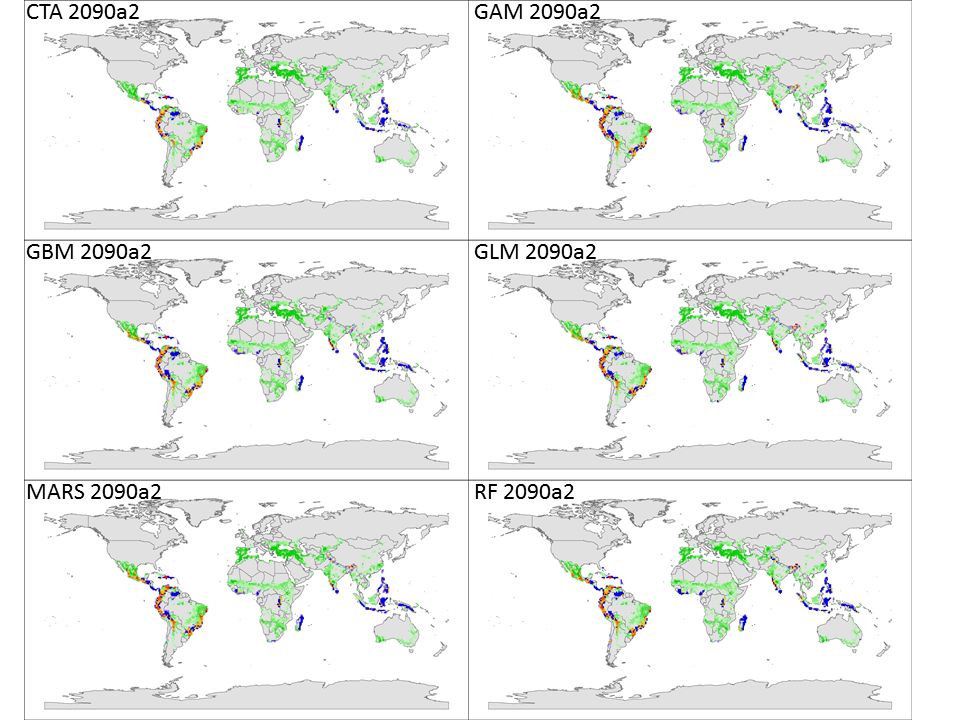

Supplement: Figure S6 — Global map of crop suitability and suitability for range restricted birds change in 2090 under A2 emission scenario using 6 Species Distribution Model results. Areas in which overall decreases are anticipated in crop suitability are shown in green, with increasing color intensity indicating multiple GCM agreement. Areas of declining climatic suitability for restricted range birds are shown in blue, with increasing color intensity indicating multiple GCM agreement. Overlap of declining crop suitability and declining restricted range bird climatic suitability is shown in shades of yellow (lowest GCM agreement) to red (highest GCM agreement). (TIF) [file pone.0072590.s007.tif]
